# The role of *Fusobacterium nucleatum* outer membrane vesicles in disease and related mechanisms: a scoping review of pathogenesis and potential therapeutic applications

**DOI:** 10.1080/20002297.2026.2717775

**Published:** 2026-09-23

**Authors:** Chunfeng Luo, Zhiwen Li, Fengyang Yi, Ping Wen, Ting Zou, Shan Jiang, Zuolin Jin

**Affiliations:** a Shenzhen Clinical College of Stomatology, Southern Medical University, Shenzhen, People's Republic of China; b Central Laboratory, Shenzhen Stomatology Hospital (Pingshan) of Southern Medical University, Shenzhen, People's Republic of China; c Department of Stomatology, Shenzhen Maternity and Child Healthcare Hospital, Southern Medical University, Shenzhen, Guangdong Province, People's Republic of China

**Keywords:** *Fusobacterium nucleatum*, outer membrane vesicles, inflammatory bowel disease, colorectal cancer, virulence factors, intestinal barrier

## Abstract

**Objective:**

*Fusobacterium nucleatum* (*F. nucleatum*) has been implicated in the exacerbation of cancer development and metastasis. Outer membrane vesicles (OMVs) derived from Gram-negative bacteria have gained considerable attention as a message bridge between bacteria and host cells. This review aims to comprehensively explore the role of *F. nucleatum* OMVs in the pathogenesis of diseases, their interaction with host cells, and their potential as novel therapeutic targets or vector tools.

**Design:**

A comprehensive literature search was conducted in PubMed, Scopus, and Web of Science for studies published up to March 2026. Subsequently, the review was performed in accordance with the PRISMA-ScR guidelines.

**Results:**

Twenty-one studies were finally included in accordance with the predefined inclusion criteria. These studies suggest that OMVs derived from *F. nucleatum* contain multiple virulence factor proteins, including FadA, Fap2, and FomA, and are associated with inflammatory bowel disease, colorectal cancer, oral squamous cell carcinomas, and rheumatoid arthritis. *F. nucleatum* OMVs can be internalized by intestinal epithelial cells, leading to the downregulation of tight junction proteins. These vesicles also induce the release of proinflammatory cytokines through the activation of autophagy, the NF-кB signaling pathway, or the AIM2 inflammasome. Additionally, they promote macrophage polarization towards a proinflammatory phenotype, ultimately triggering a cascade of inflammatory responses. Furthermore, *F. nucleatum* OMVs enhance bacterial adhesion and autoaggregation, facilitate immune suppression, and promote tumor growth and metastasis. Notably, *F. nucleatum* OMVs also possess potential as engineered drug delivery vehicles for cancer immunotherapy.

**Conclusion:**

This review summarizes the current evidence regarding the molecular mechanisms through which *F. nucleatum* OMVs interact with both immune and non-immune cells, as well as the identification and composition of these OMVs, providing insights into their potential for preventive and therapeutic applications.

## Introduction

*Fusobacterium nucleatum* (*F. nucleatum*) is a Gram-negative obligate anaerobe commonly found in the oral cavity, as well as in the urogenital, intestinal, and upper digestive systems [1,2]. *F. nucleatum* can coaggregate with various microbial species in the oral cavity to form dental plaque [3] and binds to a variety of host cells by colonisation and invasion, primarily relying on adhesins, including Aid1, CmpA, Fap2, FomA, FadA, and RadD [2]. *F. nucleatum* serves as a bridge between primary colonisers (such as *Streptococcus* species) and anaerobic secondary colonisers (such as *P. gingivalis*) in dental plaque biofilm, facilitating the establishment of anaerobic microenvironments that promote coaggregation of strict anaerobes [4,5]. Additionally, it can be recognised by the pattern recognition receptors (PRRs) of host cells, triggering various immune responses and exacerbating inflammation, thereby promoting tumorigenesis by modulating the tumour-immune microenvironment [6].

Several known virulence factors are present in *F. nucleatum*, which are associated with inflammation and oncogenesis, including the FadA adhesin, Fap2, and FomA [7]. One study has revealed that the levels of the FadA gene in the colon tissue from patients with adenomas and adenocarcinomas are significantly higher compared to those in normal individuals. The binding of FadA to E-cadherin activates *β*-catenin signalling, differentially regulating inflammatory and oncogenic responses in colorectal cancer (CRC) cells [8]. Fap2 mediates fusobacterial CRC enrichment by binding to the carbohydrate moiety Gal-GalNAc, which is overexpressed in human colorectal adenocarcinoma and metastases [9]. In addition to Fap2, FomA, a major outer membrane pore protein of *F. nucleatum*, plays a role in helping the bacterium evade host immune surveillance [10]. *F. nucleatum* can also secrete outer membrane vesicles (OMVs) that carry its virulence factors, thereby exerting its pathogenicity. Typically, a portion of the outer membrane separates from the bacterial surface and encloses components of the periplasmic space to form OMVs.

OMVs are nano-sized, spherical, bilayered vesicles derived from both pathogenic and non-pathogenic Gram-negative bacteria, ranging in size from 20 to 300 nm. They were first described more than 50 years ago [11]. These vesicles carry multiple bacteria-derived components, including phospholipids, lipopolysaccharide (LPS), outer membrane proteins (OMPs), periplasmic proteins, DNA, RNA, and peptidoglycan [12]. Due to their composition, OMVs are involved in multiple physiological processes, including the transport of virulence factors, nutrient acquisition, intercellular communication, and modulation of immune system responses [13]. Moreover, OMVs contain a higher concentration of virulence factors compared to their parent bacteria, and their outer membrane structures protect virulence proteins in the host environment, facilitating long-distance delivery to various organs and tissue targets via the circulation [14].

Intriguingly, accumulating evidence suggests that OMVs derived from *F. nucleatum* are not only important virulence mediators but also play critical roles in the pathogenesis of a broad spectrum of inflammatory diseases and malignancies through long-distance dissemination and modulation of host immune responses [15]. Among these inflammatory diseases, periodontitis is the most representative oral disease. Periodontitis is a chronic inflammatory disease that damages the tissues surrounding the teeth, manifesting as gingival bleeding, alveolar bone loss, and tooth mobility. Its initiation and progression are driven by a dysbiosis of the commensal oral microbiota (dental plaque), which interacts with the host immune defence system, aggravating inflammatory responses [16]. Moreover, among the microorganisms associated with periodontal dysbiosis, *F. nucleatum* is considered a key bridging pathogen that facilitates multispecies biofilm formation and promotes periodontal inflammation together with other major periodontal pathogens, including *Porphyromonas gingivalis, Treponema denticola,* and *Tannerella forsythia* [17]. Further evidence indicates that *F. nucleatum* OMVs contribute directly to periodontal tissue destruction by aggravating inflammation, enhancing osteoclastogenesis, and accelerating alveolar bone loss in an animal model of periodontitis [18].

Inflammatory bowel disease (IBD), including ulcerative colitis (UC) and Crohn’s disease (CD), is a chronic relapsing inflammatory disorder of the gastrointestinal tract [1,19]. *F. nucleatum* is enriched in the intestinal mucosa of patients with IBD and contributes to disease progression by promoting intestinal inflammation and disrupting epithelial barrier function. Recent studies further demonstrate that *F. nucleatum* OMVs mediate these pathogenic effects by inducing epithelial cell death, oxidative stress, and aberrant immune responses, thereby exacerbating intestinal injury and inflammation [20]. Furthermore, periodontal *F. nucleatum* infection has been implicated in rheumatoid arthritis (RA), where circulating OMVs may disseminate proinflammatory virulence factors to distant tissues, trigger aberrant immune activation, and promote synovial inflammation and joint destruction [21,22]. These observations suggest the potential of *F. nucleatum* OMVs as key mediators linking oral microbial dysbiosis with both local and systemic diseases.

In addition to inflammatory disorders, accumulating evidence has also linked OMVs derived from *F. nucleatum* to multiple malignancies. Among these malignancies, colorectal cancer (CRC) is the most common malignancy and is closely associated with gut microbiota dysbiosis. *F. nucleatum* is frequently enriched in CRC tissues, and its OMVs play an important role in tumour progression and modulation of the tumour microenvironment [23–25]. Similar enrichment of *F. nucleatum* has also been observed in other cancers, including oral squamous cell carcinoma (OSCC) and pancreatic cancer [26,27], suggesting its broad involvement in tumorigenesis across multiple cancer types.

Despite their pathogenic effects, OMVs have emerged as promising therapeutic platforms owing to their intrinsic immunogenicity, nanoscale architecture, and amenability to genetic and biochemical engineering [28]. For example, *Escherichia coli* OMVs, as classic biovesicles with mature mass production systems, have been shown to enhance the infiltration and activation of cancer antigen-specific CD8 T cells, which exhibited high expression of TCF-1 and PD-1, leading to synergistic anti-tumour activity in combination with anti-PD-1 antibody immunotherapy [29]. In addition, OMVs can be further developed as cancer vaccine platforms through surface modification and cargo engineering, enabling enhanced tumour targeting and improved therapeutic specificity, as well as potential reversal of drug resistance [30].

Furthermore, recent studies have demonstrated that OMVs secreted by *F. nucleatum* carry virulence factors inherited from the parent bacteria, exerting pathogenic effects on local tissues as well as distant organs via systemic circulation. These findings highlight the substantial biomedical relevance and translational potential of *F. nucleatum* OMVs. A growing body of evidence suggests that *F. nucleatum* OMVs may independently contribute to disease pathogenesis. Nevertheless, comprehensive and systematic reviews focusing specifically on *F. nucleatum* OMVs remain scarce. To address this gap, the present review systematically summarises existing research on *F. nucleatum* OMVs, with particular emphasis on recent advances, underlying mechanisms of action, and their therapeutic applications in modulating various diseases. In addition, we review the isolation and identification methods, as well as the structural and compositional characteristics of *F. nucleatum* OMVs, aiming to highlight their potential as cutting-edge tools for biomedical research and clinical translation.

## Methods

### Protocol

This review adhered to the guidelines outlined by the Preferred Reporting Items for Systematic Reviews and Meta-Analyses extension for Scoping Reviews (PRISMA-ScR) [31].

### Search strategy and study selection

The literature search was performed in three electronic databases: Scopus, Web of Science, and PubMed, to identify articles related to *F. nucleatum* OMVs up to March 2026. Additionally, two authors independently conducted a manual search to identify further relevant studies. The search strategy utilised Boolean operators (AND, OR) to combine the following keywords: (‘*Fusobacterium nucleatum’* OR *F. nucleatum* OR Fn) AND (‘outer membrane vesicles’ OR OMV OR ‘extracellular vesicle’ OR EV).

### Eligibility and exclusion criteria

The eligibility criteria were established to reflect the objective of this scoping review, which was to map the current evidence regarding the biological functions and roles of *F. nucleatum* OMVs in diseases. Studies were included if they met the following criteria: 1) investigated *F. nucleatum* OMVs as the primary research focus, including in vitro studies, in vivo studies, and clinical studies. 2) examined their biological characteristics, mechanisms of action, pathogenic effects, or therapeutic applications in diseases. 3) original, peer-reviewed, and published in English up to March 2026. 4) reported clear titles and abstracts, along with sufficient information about the methodology and results.

Studies were excluded if they were: (1) reviews, systematic reviews, meta-analyses, case reports, editorials, book chapters, conference abstracts, or letters to the editor; (2) published in languages other than English; or (3) not focused on *F. nucleatum* OMVs.

### Study selection

The study selection process is shown in Figure 2. Two reviewers independently screened the titles and abstracts of the retrieved articles to determine their relevance to *F. nucleatum* OMVs. Then the articles that were not excluded underwent a full-text review and were assessed based on the eligibility criteria. In case of any conflict, a third reviewer resolved the disagreement.

### Data synthesis and extraction

Data from the included studies were extracted in a standardised manner, including (1) Author(s); (2) Year of publication; (3) OMV-isolating strain; (4) OMVs isolation methods; (5) In vivo model (e.g. animal model): OMVs treatment; (6) In vitro model (e.g. cells/cell lines): OMVs treatment; (7) Impacted key molecules/pathways; and (8) Main mechanism and key findings. A narrative synthesis approach was employed to summarise and interpret the findings from the included studies.

### Risk of bias assessment

In this review, we used the QUIN tool, which contains 12 criteria, to evaluate the risk of bias for the in vitro studies, as shown in Table 1 [32]. Each criterion can be scored as 2 (adequately specified), 1 (inadequately specified), 0 (not specified), or not applicable (exclude criteria from calculation). The final score for a specific in vitro study was calculated by the following formula: Final score = (Total score × 100)/(2 × number of criteria applicable). A final score above 70% indicates a low risk of bias, a score between 50-70% indicates a medium risk, and a score below 50% indicates a high risk of bias.

The SYRCLE risk of bias tool was employed for in vivo studies, which contains 10 items related to selection bias, performance bias, detection bias, attrition bias, reporting bias, and other sources of bias [33]. Each item was judged as follows: ‘yes’ indicating low risk of bias, ‘no’ indicating high risk of bias, and ‘unclear’ assigned when available information was insufficient for assessment. Risk of bias assessments were conducted by two reviewers, with any disagreements resolved through consensus or by consulting a third reviewer. The outcomes of the SYRCLE risk of bias evaluation are presented in Figure 1.

**Table 1.** Assessment of risk of bias in in vitro studies using the QUIN tool.

Author(year)	D1	D2	D3	D4	D5	D6	D7	D8	D9	D10	D11	D12	Total score	Risk of bias
Chen et al. [18]	2	0	1	2	2	1	0	2	1	0	2	2	62.50%	Medium
Chen et al. [34]	2	0	1	2	2	0	1	2	0	0	2	2	58.33%	Medium
Engevik et al. [35]	2	0	1	2	2	0	1	2	1	1	2	2	66.66%	Medium
Martin-Gallausiaux et al. [36]	2	0	2	1	2	0	NA	2	0	1	2	2	63.64%	Medium
Hong et al. [21]	2	1	2	2	2	0	1	2	2	2	1	2	79.17%	Low
Lamprina et al. [37]	2	1	2	2	2	0	1	2	2	2	1	2	79.17%	Low
Li et al. [38]	2	0	1	2	2	0	1	2	1	0	2	2	62.50%	Medium
Li et al. [39]	2	0	1	2	2	1	1	2	1	1	2	2	75.00%	Low
Lin et al. [40]	2	0	1	2	1	0	2	2	0	0	1	2	50.00%	Medium
Liu et al. [41]	2	0	2	2	2	0	NA	2	0	0	1	2	59.10%	Medium
Liu et al. [42]	2	0	2	2	2	0	2	2	0	0	1	2	62.50%	Medium
Liu et al. [43]	2	0	2	2	2	0	NA	2	0	0	1	2	59.10%	Medium
Qi et al. [44]	2	0	2	2	2	0	NA	2	0	0	1	2	59.10%	Medium
Shangguan et al. [45]	2	0	1	2	2	0	1	2	1	1	2	2	66.67%	Medium
Shen et al. [46]	2	0	1	2	2	0	1	2	1	1	2	2	66.67%	Medium
Wang et al. [47]	2	0	2	2	2	0	NA	2	0	0	1	2	59.10%	Medium
Wei et al. [48]	2	0	2	2	2	0	NA	2	0	0	1	2	59.10%	Medium
Zhang et al. [49]	2	0	2	2	2	1	NA	2	0	0	2	2	68.18%	Medium
Zhang et al. [17]	2	0	1	2	2	0	0	2	0	1	2	2	58.33%	Medium
Zhao et al. [50]	2	1	2	2	2	0	1	2	2	2	2	2	83.33%	Low
Zheng et al. [51]	2	1	2	2	2	1	1	2	2	2	2	2	87.50%	Low

D1: Clearly stated aims/objectives; D2: Detailed explanation of sample size calculation; D3: Detailed explanation of sampling technique; D4: Details of comparison group; D5: Detailed explanation of methodology; D6: Operator details; D7: Randomisation; D8: Method of measurement of outcome; D9: Outcome assessor details; D10: Blinding; D11: Statistical analysis; D12: Presentation of results. NA: not applicable.

**Figure 1.**
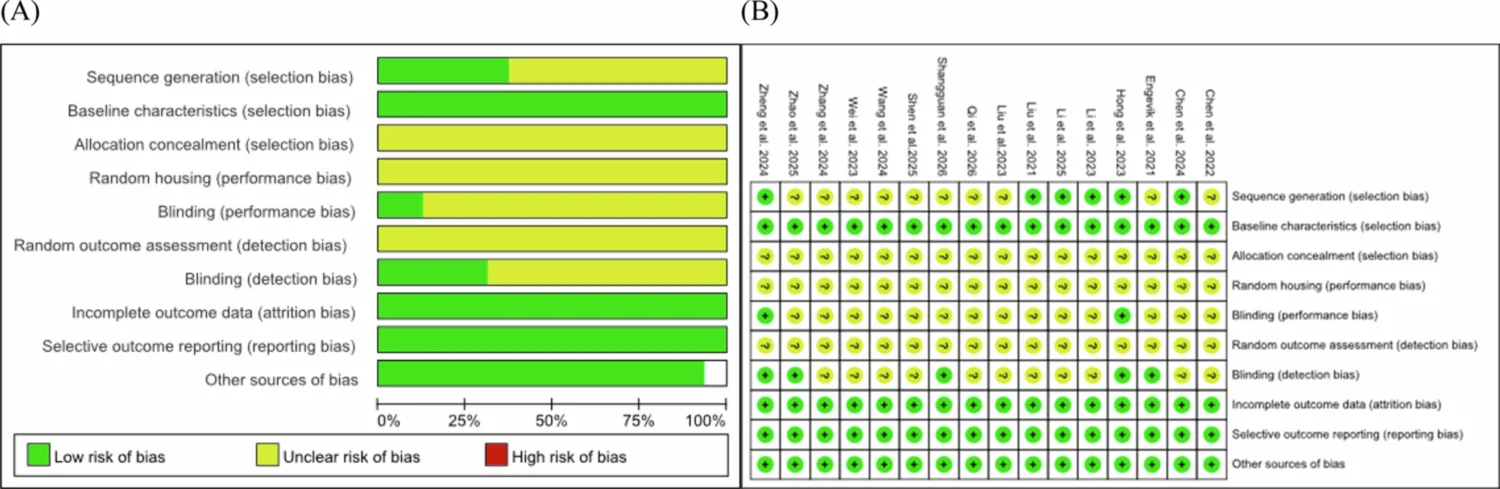
Assessment of risk of bias in in vivo studies using SYRCLE. (A) risk-of-bias graph; (B) risk-of-bias summary.

## Results

### Literature search results

The PRISMA flow chart in Figure 2 summarises the study selection process for this review. The diagram offers a comprehensive overview of the number of records identified, screened, assessed for eligibility, and ultimately included in this review, along with the reasons for exclusion. The initial search across three literature databases identified a total of 163 studies: 34 from PubMed, 50 from Scopus, and 79 from Web of Science. After removing duplicate records, 90 titles and abstracts remained for screening. Subsequently, 42 publications were evaluated through full-text reading. Of these, 10 publications were excluded during the eligibility assessment because they were reviews, and 3 were disregarded as they were written in Chinese. Finally, 21 publications met the inclusion criteria.

**Figure 2.**
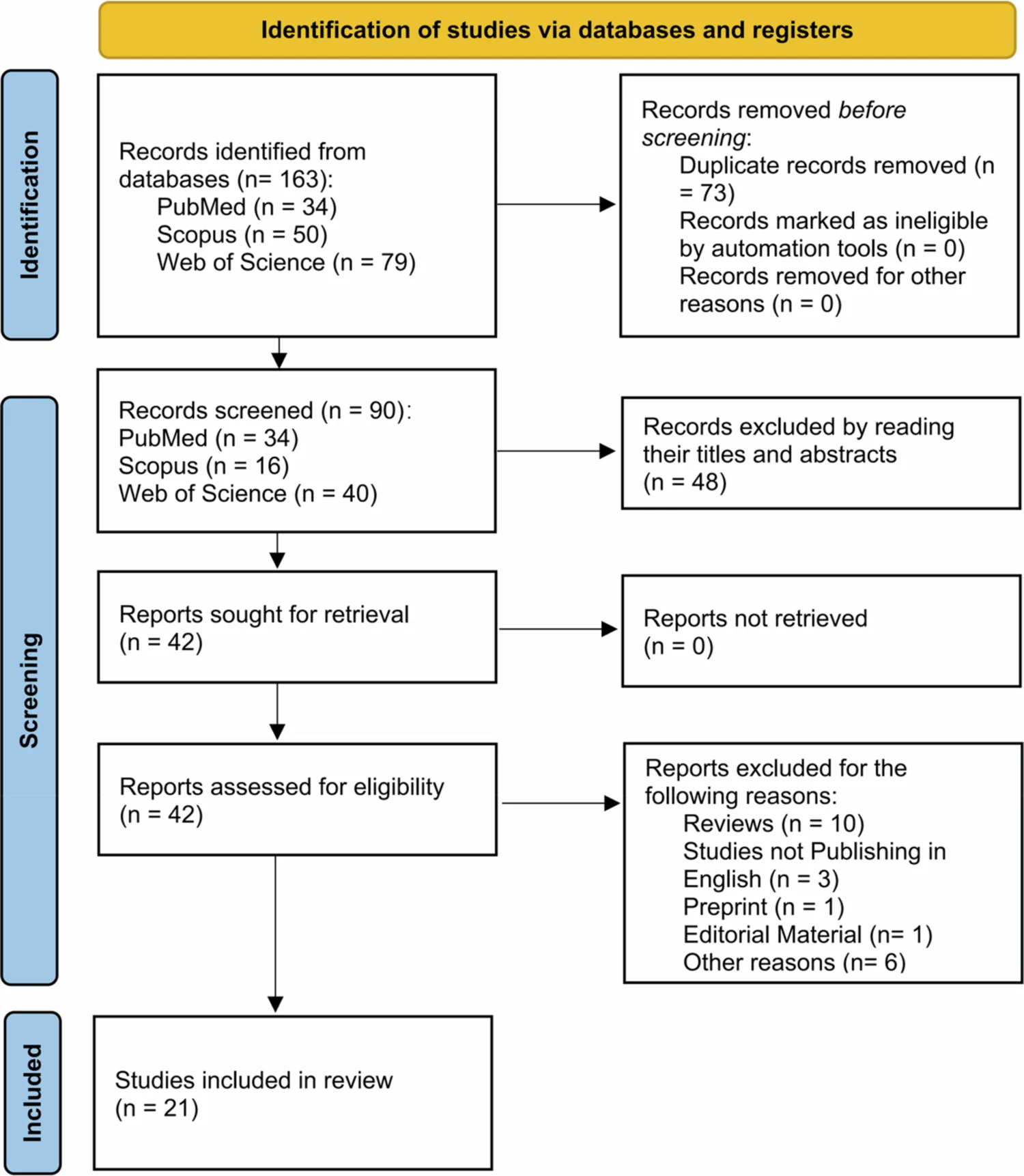
PRISMA flow chart presenting the search strategy of this review.

### Characteristics of the included studies

A total of 21 studies were ultimately identified as eligible for inclusion in this scoping review. As summarised in Table 4, the majority reported the full genus, species name, and strain number of *F. nucleatum*, whereas 6 studies failed to provide these details. Among the 21 studies, only one did not disclose the OMV isolation and purification methodology. Furthermore, just a single study comprehensively reported both the initial bacterial dosage used for OMVs production and the corresponding yield quantified via total protein concentration or nanoparticle enumeration. Notably, no clinical studies met the inclusion criteria; all included studies employed in vitro or in vivo models. Tables 2 and 3 outline the characteristics of the included studies. Three studies involved in vitro models exclusively, comprising cell cultures or established cell lines, with two utilising pre-established lines and one employing human-derived samples. The remaining 16 studies incorporated both in vivo and in vitro models.

Given the heterogeneous research objectives across eligible studies, notable discrepancies are observed in the selection of cellular sources for in vitro assays, animal model design, and experimental protocols applied to investigate *F. nucleatum* OMVs. Substantial inter-study variation also exists in core experimental parameters, encompassing OMVs dosage, stimulation concentration, and treatment duration. Additionally, primary outcome indicators differ significantly with respect to measurement dimensions, detection methodologies, and biological clinical relevance. Such prominent methodological heterogeneity renders quantitative pooled analytical approaches, most notably meta-analysis, inappropriate for integrated data synthesis and comparative interpretation. Accordingly, this review adopts a qualitative descriptive synthesis to systematically collate, contextualise, and interpret all available evidence concerning *F. nucleatum* OMVs.

**Table 2.** The summary of included studies only involved in vitro models.

Author (year)	In vitro model (e.g. cells/cell line, strains): OMVs treatment	Impacted key molecules/pathways	Main mechanism and key findings
Martin-Gallausiaux et al. [36]	HCT116 (ATCC), HT-29 (ECACC): co-culture, 5 μg/ml; Caco-2 (ATCC): co-culture, 10 μg/ml; T84 (ATCC): co-culture, 5 μg/ml	NF-кB↑, IL-8↑, TLR2↑	*F. nucleatum* OMVs induce NF-кB activation in a manner dependent on the TLR2 expression level in IECs. And OMVs treated with the competitive inhibitor peptide (‘anti-FomA’) reduce NF-kB activation compared to untreated OMVs. Pre-incubation of T84 cells with dynasore, a GTPase dynamin inhibitor, blocks dynamin-mediated membrane trafficking, leading to a reduction in NF-kB activity. *F. nucleatum* OMVs induce innate immunity of IECs by promoting NF-kB activation through dynamin-dependent endocytosis.
Lamprina et al. [37]	monocyte-derived dendritic cell (moDC): co-culture, 10 μg/ml; macrophage (moMφ): co-culture, 1 or 10 μg/ml	IL-10↑, CD80↑, TNFα↓, CD86↓	*F. nucleatum* OMVs bind to Siglec-7 at levels comparable to LPS. In moMφs, OMVs upregulate the expression of IL-10 and CD80 to a similar extent as LPS. In moDCs, OMVs deregulate the expression of CD86 and induce the production of TNFα. *F. nucleatum* OMVs induce a proinflammatory phenotype in moMφs while promoting a tumour-associated phenotype in moDCs.
Liu et al. [41]	N/A	N/A	Proteomic analysis of *F. nucleatum* OMVs revealed that they are predominantly composed of outer membrane proteins, which account for 72% of the total protein content. Additionally, 17% of the proteins have subcellular localisation traits, and 6% are periplasmic proteins. And secretion proteins are the major component of the *F. nucleatum* OMVs. *F. nucleatum* OMVs have strong immunogenic potential, as their proteins contain predicted B-cell and T-cell epitopes.
Lin et al. [40]	Human CRC cell lines Caco-2: co-culture, 1 × 10^8^ particles/mL	N/A	In Caco-2 cells, *F. nucleatum* OMVs increase mitochondrial fusion, whereas pennogenin 3-O-beta-chacotrioside, polyphyllin VI, and saponin I attenuate this effect. In a wound-healing assay, OMVs enhance the cell migration of Caco-2 cells by approximately 20%, but these compounds inhibit wound closure. Active compounds isolated from *P. polyphylla* counteract the pro-tumorigenic effects of *F. nucleatum* OMVs in a colorectal cancer model.
Zhang et al. [49]	N/A	N/A	Acidic pH alters the proteome of *F. nucleatum* OMVs. Compared with OMVs produced under neutral-pH conditions, aOMVs exhibit a 23% increase in production, with approximately 70% of the expression of OMV proteins altered. Virulence factors are upregulated. Fatty acid synthesis proteins increase, and butyrate synthesis proteins decrease.

Abbreviations: IECs, human intestinal epithelial cells. moMφs, human monocyte-derived macrophages. moDCs, human monocyte-derived dendritic cells. *P. polyphylla*, *Paris polyphylla*. aOMVs, acidic outer membrane vesicles. N/A, not available.

**Table 3.** The summary of included studies involved in vivo and in vitro models.

Author (year)	In vivo model (animal model): OMVs treatment	In vitro model (e.g. cells/cell line, strains): OMVs treatment	Impacted key molecules/pathways	Main mechanism and key findings
Chen et al. [18]	C57BL/6 wild-type mice (6 weeks old, male, 20 g body weight): the maxillary molar teeth via the silk threads injection, 1 μg/ml	Mouse gingival fibroblasts (MGFs): co-culture; Mouse macrophage cell line RAW264.7 (ATCC TIB71): co-culture, 24 h; Bone Marrow-Derived Macrophages (BMDMs): co-cultured, 1 μg/mL, 24 h	TNF-α↑, iNOS↑, phosphorylation of p65↑, ROS↑, LDH↑	*F. nucleatum* OMVs can stimulate BMDMs to adopt morphological characteristics similar to M1 macrophages. OMVs upregulate the expression of TNF-*α* and iNOS, as well as phosphorylation of p65. OMVs drive polarisation of M0-like macrophages toward proinflammatory M1-like phenotype, accompanied by elevated ROS levels. In MGF cells, OMVs co-cultured with M1 macrophages induce apoptosis and enhance the release of LDH. In a mouse model, OMVs increase the number of osteoclasts, thereby promoting the occurrence and development of periodontitis. *F. nucleatum* OMVs promote the polarisation of macrophages toward the proinflammatory M1 phenotype, thereby exacerbating the toxicity of OMVs on MGFs, contributing to the development of periodontitis.
Chen et al. [34]	BALB/c nude mice (20 g, 4 weeks old): intratumor injection, 1 mg/kg, once a week	CAL27 and HSC3 human HNSCC cell lines: co-culture, 5 μg/mL, 24 h	E-cadherin↓, Vimentin/N-cadherin↑	*F. nucleatum* OMVs decrease E-cadherin expression while increasing the expression of vimentin and *N*-cadherin in CAL27 and HSC3 cells. OMVs also increase LC3B expression in the cells and induce activation of autophagy pathways. In the tumour-bearing mouse model, *F. nucleatum* OMVs can promote lung metastasis, potentially through the induction of the EMT phenotype in cancer cells.
Engevik et al. [35]	C57BL/6 mice (10 to 16 weeks)	Human colon cell line HT-29 (ATCCHTB-38): co-culture, 5% purified OMVs; human stem cell-derived colon line C103	TLR4↑→NF-кB↑, IL-8↑,	*F. nucleatum* OMVs activate TLR4, leading to increased secretion of IL-8 and TNF and activation of the NF-кB signalling pathway in human colon cells. *F. nucleatum* OMVs may contribute to the development of intestinal inflammation.
Hong et al. [21]	DBA1/J mice (eight-ten-week-old male), C57 BL/6 WT mice (eight-ten-week-old male), Rab5a-/- mice (on a C57BL/6 background): Gavage, 100 μg/kg	Bone Marrow-Derived Macrophages (BMDMs)	Rab5a-YB-1 axis↑ FadA	*Fusobacterium nucleatum* enriched in the RA gut exacerbates arthritis through OMVs delivering FadA to joints. FadA activates the Rab5a-YB-1 axis in synovial macrophages, triggering synovitis.
Li et al. [38]	BALB/c nude mice (female, 6–8 weeks, 18-22 g): intratumor injection 100 μg/mL or tail vein injection 100 μg	BC cell lines (MDA-MB-231 and MCF-7): co-culture, 10 μg	TLR4↑	*F. nucleatum* OMVs enhance the proliferation, migration, and invasion capacity of BC cells through activation of the TLR4 pathway. In the BALB/c nude mice xenograft model, OMVs induced tumour growth and liver metastasis, effects that are attenuated by TLR4 silence. *F. nucleatum* OMVs promote breast cancer growth and metastasis via TLR4-dependent signalling.
Li et al. [39]	C3H/He mice and NOD-SCID mice (4–6 weeks old): intratumor injection, 10 μg	Human head and neck squamous cell carcinoma cell lines HN6 and SCC9, mouse head and neck squamous cell carcinoma cell line SCC7, and human monocyte leukaemia cell line THP-1: co-culture, 1 μg/mL	TDO2/AHR↑→TAMs↑	F. nucleatum OMVs are internalised by TAMs. OMVs carry tryptophanase, which hydrolyses tryptophan into indole, activating the TDO2/AHR pathway. This upregulates immunosuppressive cytokines and immune checkpoints, thereby reducing cytotoxic T lymphocyte infiltration.
Liu et al. [42]	C57BL/6 mice (males, 8-week-old): intragastric administration, 10 or 50 μg/day	peripheral blood mononuclear cell (PBMC)-derived macrophages, human colonic epithelial cell line Caco-2 cells: co-culture, 1 μg/ml	TNF-*α*, iNOS, and ROS↑; IL-10↓, RIPK1 and RIPK3↑, ZO-1, Claudin-1, occluding↓, mucin1 and mucin2↑, FADD-RIPK1-cCASP-3 signalling pathway↑	In macrophages, *F. nucleatum* OMVs increase the expression of TNF-*α*, iNOS, and ROS while deregulating the levels of IL-10. These changes indicate that OMVs promote macrophage polarisation into the proinflammatory M1 phenotype, inducing oxidative damage in the cell. In Caco-2 cells, OMVs upregulate RIPK1 and RIPK3 expression, leading to necrosome formation, necroptosis, and impaired epithelial barrier function. In DSS-induced colitis models, OMVs significantly decrease the expression of ZO-1, claudin-1, and occludin, as well as the secretion of mucin1 and mucin2, indicating disruption of epithelial barrier integrity. And OMVs activate FADD-RIPK1-cCASP-3 signalling pathway, further contributing to intestinal barrier dysfunction.
Liu et al. [43]	Mice: tail vein injection	4T1 cells	TNF-*α* and INF-γ↑, IL-6 and IL-10↓	OMV@Fe-ZIF-90@M can efficiently target tumours using *F. nucleatum* OMVs, suppressing intratumoral bacteria and inducing ICD, and significantly enhances immunogenicity. Furthermore, OMV@Fe-ZIF-90@M effectively induces DC maturation and T-cell infiltration, increasing the levels of TNF-*α* and IFN-*γ* while decreasing IL-6 and IL-10 levels, thereby converting cold tumours into immune-activated microenvironments.
Qi et al. [44]	Wistar rats (male, ~200 g): local injection, 0.1 mL -ABSI per rat	L929 fibroblasts, human gingival fibroblasts: co-culture, 150 μg/mL -ABSI; *S. gordonii*, *F. nucleatum*, *P. gingivalis*, and *L. reuteri*	ROS↑	OMV-AgBiS_2_@ICG (OMV-ABSI) exhibits excellent photodynamic and photothermal therapeutic capabilities, along with second near-infra-red (NIR-II) imaging functionality. It targets periodontal pathogens by leveraging the natural adhesins on *Fusobacterium nucleatum* OMVs, accelerating electron transfer, which enhances oxidative stress and promotes the accumulation of endogenous ROS. This process, in turn, inhibits DNA transfer and causes damage to membranes, proteins, lipids, and DNA.
Shangguan et al. [45]	BALB/c nude mice: intratumor injection	Human bladder cancer cell lines T24 (RRID: CVCL_0554); SW780 (RRID: CVCL_5G49); Immortalised urothelial cell line SV-HUC1 (RRID: CVCL_3798); Human lymphatic endothelial cells (HLECs, Cat. No. 2500, RRID: CVCL_1H44)	TLR2, NF-кB pathway, IL-6↑	Proteomic analysis results indicate that the most abundant component of *F. nucleatum* OMVs is the outer membrane protein FomA. FomA-containing OMVs can interact with TLR2, activating the TLR2/NF-кB/IL-6 signalling axis in BCa cells. Elevated expression of IL-6 facilitates the transformation of macrophages into an M2b phenotype, thus enhancing VEGF-C expression and lymph node metastasis in BCa.
Shen et al. [46]	Balb/c mice (6–8 weeks, female)	4 T1 cells	GPX4↓, P–P65↑, IL-12, and TNF-α↑	FMV@PCFPC downregulates GPX4 expression in tumour cells, suggesting its potential to induce tumour cell death via the ferroptosis pathway. Furthermore, it upregulates *P*-P65 phosphorylation, indicating activation of the TLR4–NF-κB signalling pathway, and triggers the secretion of IL-12 and TNF-*α* from macrophages. FMV@PCFPC induces dual ferroptosis in both bacteria and tumour cells to alleviate immunosuppression and activate ICD. This process promotes DC maturation, facilitates the activation and infiltration of cytotoxic T cells, and stimulates cytokine secretion, thereby improving the efficacy of tumour immunotherapy.
Wang et al. [47]	BALB/c (6–8 weeks old, female, 18–20 g): intratumoral injection, 4T1: 5 μg; 4MOSC1: 1 μg	murine breast cancer cell line 4T1 (Cat CRL-2539) and 4MOSC1 cells: co-culture, 1 μg ml^−1^	GSDMD, GSDME, and MLKL↑	*F. nucleatum* OMVs enhance the expression level of PANoptosis execution proteins by modulating their ubiquitination, including the GSDMD, GSDME, and MLKL proteins in tumour cells. Treatment with OMVs in combination with oHSV induces higher expression levels of PANoptosis executioners. In tumour tissue, the combination treatment of OMVs and oHSV increases the fraction of mature DCs and CD8 + T cell infiltration while promoting the conversion of M2 TAMs into M1 phenotype.
Wei et al. [48]	C57BL/6J CARD3 knockout (CARD3^–/–^) mice (male, 6 weeks old) and C57BL/6J wild-type (CARD3^wt^) mice (male, 6 weeks old): intragastric administration, 100 μg/day in 200 μL PBS per mouse	IECs (NCM460 and Caco-2): co-culture, 100 μg per mouse	IL-1β, IL-6, and TNF-α↑; IL-10↓, ZO-1 and occludin↓	*F. nucleatum* OMVs can be internalised by IECs to enhance cellular inflammatory responses, including upregulation of IL-1β, IL-6, and TNF-*α*, and disrupt intercellular tight junction proteins, such as ZO-1 and occludin. And OMVs decrease p62 and increase LC3II protein levels, effects that can be reversed by CQ, an autophagy lysosomal inhibitor. In Caco-2 monolayer cells, OMV-induced reductions in TEER values and increased FD4 permeability can be restored with CQ pretreatment. In IECs co-cultivated with *F. nucleatum* OMVs, autophagy is reduced in cells overexpressing miR-574-5p and/or CARD3 knockdown. In DSS-induced colitis mouse models, the protective effect of miR-574-5p on Fn-OMV-induced colitis depends on its downstream target gene CARD3. *F. nucleatum* OMVs promote experimental colitis, epithelial barrier damage, and autophagy activation via the miR-574-5p/CARD3 axis.
Zhang et al. [17]	SD rats (5 weeks old, male): injection in mesial and distal periodontal tissues, 1 μg/μl, three times a week	HPDLSCs: co-culture, 0, 5, 15, and 30 μg/ml	NFкB↑→NLRP3↑ CTSK↑, DCST1↑	*F. nucleatum* OMVs induce periodontitis. In a mouse model, OMVs cause alveolar bone loss, tissue destruction, inflammation (elevated NLRP3, IL-1β, IL-18, and TNF-*α*), and osteoclast activation. In hPDLSCs, OMVs enter them through caveolin-dependent endocytosis, activate the NF-κB and NLRP3, causing cytokine release and ROS, and suppress osteogenic mineralisation.
Zhao et al. [50]	C57BL/6 mice (male, six-week-old): intragastric administration, 15 μg in 200 μL saline for each mouse	Human normal colon epithelial cell line NCM460: co-culture, 1 μg/mL	AIM2↑→ GSDMD and IL-1β↑; ZO-1 and occludin↓	In IECs, *F. nucleatum* OMVs induced pyroptosis through the AIM2 inflammasome. In DSS-induced mouse models, *F. nucleatum* OMVs induce the expression of AIM2 inflammasome-dependent pyroptosis-related proteins in colon tissue, including GSDMD and IL-1β. And OMVs decrease the expression of ZO-1 and occludin. However, these effects can be restored by treatment using A151 and DNase I. *F. nucleatum* OMVs, carrying the virulence factor DNA, are internalised by IECs, triggering AIM2 inflammasome-dependent pyroptosis and intestinal barrier disruption.
Zheng et al. [51]	C57BL/6J mice (male, 8 weeks old, at least 20 g in weight): oral or tail intravenous injection, 1 μg	Human CRC cell lines DLD-1: co-culture, 5 μg/ml	FomA	*F. nucleatum* OMVs transfer the outer membrane protein FomA to the cell surface by fusing with CRC cells. FomA then acts as an adhesion target for *F. nucleatum* by interacting with FN1441, promoting bacterial autoaggregation and niche formation.

Abbreviations: BMDMs, bone marrow-derived macrophages. iNOS, inducible nitric oxide synthase. ROS, reactive oxygen species. MGFs, mouse gingival fibroblasts. LDH, lactate dehydrogenase. EMT, epithelial-mesenchymal transition. RA, rheumatoid arthritis. TAMs, tumour-associated macrophages. TDO2/AHR, tryptophan-2,3-dioxygenase 2/aryl hydrocarbon receptor. ICD, immunogenic cell death. BCa, bladder cancer. oHSV, HSV-1-based oncolytic viruses. IECs, intestinal epithelial cells. hPDLSCs, human periodontal ligament stem cells. CRC, colorectal cancer. DC, dendritic cell. DSS, dextran sulphate sodium. BC, breast cancer.

### Risk of bias assessment

Using the QUIN tool to evaluate the methodological quality of in vitro studies, all studies exhibited medium or low risk of bias. This indicates a generally robust study design and conduct. However, the SYRCLE risk of bias assessments for the included in vivo studies reveal a notable variability across domains. In sequence generation, most studies showed low or unclear risks. Baseline characteristics, attrition bias, and reporting bias were well controlled, with all studies rated as low risk. However, in terms of allocation concealment, random housing, performance, and detection bias, most studies were rated as unclear, while some studies indicated a low risk. Although the overall risk of bias in in vivo studies is relatively low, there remains uncertainty regarding randomisation and blinding. These factors may limit the overall internal validity and reliability of the findings.

## Discussion

### *F. nucleatum* OMVs isolation and characterisation

OMVs are composed of various components derived from the parent bacteria and play a crucial role in the pathogenicity of various diseases [13]. These vesicles interact with host cells, facilitating inflammation and promoting cancer metastasis, and display substantial therapeutic potential for emerging treatment modalities. However, the production of OMVs can be influenced by several factors, including stress factors, the growing conditions, and different growth phases of the source bacteria [52]. Previous studies have demonstrated that the quantity, size, protein composition, pathogenicity, and immunogenicity of OMVs produced by bacteria at different stages of growth exhibit considerable variation [53,54]. Furthermore, the isolation and purification of OMVs is a prerequisite for investigating their pathogenic characteristics and developing their potential biomedical applications. Therefore, in Table 4, we have compiled a comprehensive overview of the various isolation methods for *F. nucleatum* OMVs as described in the included literature.

**Table 4.** Isolation and purification methods of *F. nucleatum* OMVs.

Author(s)	OMV-isolating strain	OMVs isolation method
Chen et al. [18]	*F. nucleatum* ATCC 25586	Combined centrifugation (centrifugation + ultracentrifugation + density gradient centrifugation), Microfiltration
Chen et al. [34]	*F. nucleatum* ATCC 25586	Combined centrifugation (centrifugation + ultracentrifugation + density gradient centrifugation), Microfiltration
Engevik et al. [35]	*F. nucleatum* subsp. *Polymorphum* ATCC 10953	Combined centrifugation (centrifugation + ultracentrifugation + density gradient centrifugation), Ultrafiltration, Ammonium sulphate precipitation
Martin-Gallausiaux et al. [36]	*F. nucleatum* subsp *nucleatum* DSM 15643 - ATCC 23726	Combined centrifugation (centrifugation + ultracentrifugation + density gradient centrifugation), Combined filtration (microfiltration + ultrafiltration)
Hong et al. [21]	*F. nucleatum* ATCC 10953	Combined centrifugation (centrifugation + ultracentrifugation), Combined filtration (microfiltration + ultrafiltration)
Lamprina et al. [37]	*F. nucleatum* ssp. *animalis* ATCC 51191	Combined centrifugation (centrifugation + ultracentrifugation + density gradient centrifugation), Combined filtration (microfiltration + ultrafiltration)
Li et al. [38]	*F. nucleatum* strain ATCC 25586	Ultracentrifugation, Microfiltration
Li et al. [39]	*F. nucleatum* ATCC 10953	Combined centrifugation (centrifugation + ultracentrifugation), Microfiltration
Lin et al. [40]	*F. nucleatum* (BCRC17680)	Combined centrifugation (centrifugation + ultracentrifugation), Microfiltration
Liu et al. [41]	*F. nucleatum* subsp. *animalis* 7-1 (EAVG_002)	Combined centrifugation (centrifugation + ultracentrifugation + density gradient centrifugation), Microfiltration
Liu et al. [42]	*F. nucleatum*	Combined centrifugation (centrifugation + ultracentrifugation + density gradient centrifugation), Combined filtration (microfiltration + ultrafiltration)
Liu et al. [43]	*F. nucleatum*	Density gradient centrifugation
Qi et al. [44]	*F. nucleatum*	Combined centrifugation (centrifugation + ultracentrifugation), Microfiltration
Shangguan et al. [45]	*F. nucleatum*	Combined centrifugation (centrifugation + ultracentrifugation), Microfiltration
Shen et al. [46]	*F. nucleatum*	Combined centrifugation (differential centrifugation + ultracentrifugation), Ultrafiltration
Wang et al. [47]	*F. nucleatum* ATCC 25586	Combined centrifugation (centrifugation + ultracentrifugation), Combined filtration (microfiltration + ultrafiltration)
Wei et al. [48]	*F. nucleatum*	Combined centrifugation (centrifugation + ultracentrifugation), Microfiltration
Zhang et al. [49]	*F. nucleatum* ATCC 23726	Combined centrifugation (centrifugation + ultracentrifugation + density gradient centrifugation), Microfiltration
Zhang et al. [17]	*F. nucleatum* ATCC 25586	Combined centrifugation (centrifugation + ultracentrifugation + density gradient centrifugation), Combined filtration (microfiltration + ultrafiltration)
Zhao et al. [50]	*F. nucleatum* ATCC 25586	N/A
Zheng et al. [51]	*F. nucleatum* ATCC 25586, ATCC 23726	Combined centrifugation (centrifugation + ultracentrifugation + density gradient centrifugation), Microfiltration

Abbreviation: N/A, not available.

#### The isolation of *F. nucleatum* OMVs

We have found that ultracentrifugation and microfiltration are the predominant techniques employed for the separation and enrichment of *F. nucleatum* OMVs. Specifically, once the bacteria culture reaches the logarithmic growth phase, the culture medium containing the bacteria is subjected to centrifugation at 6000–10,000 × g for 30 min at 4 °C to remove the bacteria and debris. The resulting supernatant is subsequently filtered through the 0.22 or 0.45 μm membrane and subjected to ultracentrifugation at 160,000–200,000 × g for 2 h at 4 °C to pellet OMVs [38–40,44,45,48]. Alternatively, ultrafiltration can be utilised in the process [21,46,47]. Additionally, some studies repeatedly perform the steps of ultracentrifugation to purify crude OMVs, which are then resuspended in sterile PBS and stored at −80 °C until downstream experiments. Other approaches, such as OptiPrep (Iodixanol) or sucrose density gradient centrifugation, are also employed in some studies to enhance the purity of the *F. nucleatum* OMVs [17,18,34–37,41–43,49,51]. In this procedure, a sample containing vesicles of varying sizes and macromolecules is layered onto the surface of a density gradient, which increases from top to bottom. During ultracentrifugation, different molecules sediment through the gradient at distinct rates, allowing for their separation based on size and density [55].

While the procedural steps of ultracentrifugation are relatively straightforward, it is time-consuming, demands high-performance equipment and stringent environmental conditions, and often results in lower overall efficiency. In contrast, microfiltration requires less sophisticated apparatus and is simpler to operate. However, it does not fully eliminate macromolecular contaminants. Ultrafiltration, which utilises 50–100 kDa ultra-centrifugal filters, offers a more effective means to remove large molecular impurities. Furthermore, many studies have employed density gradient centrifugation to further refine the OMVs, yielding higher purity fractions. This technique, however, involves multiple steps and requires specific density media, in addition to ultracentrifugation. Nevertheless, in practice applications, a combination of these methodologies is frequently employed to isolate and purify OMVs, as it enhances both the purity and homogeneity of the vesicles. This multi-step strategy holds significant promise for advancing the study of OMVs' composition and pathogenicity, thereby providing a foundation for investigating their potential as diagnostic biomarkers and therapeutic applications.

#### The characterisation of *F. nucleatum* OMVs

Although OMVs have been successfully extracted, their observation and identification before their application in subsequent experiments remain critical. Performing both qualitative and quantitative analyses of these vesicles is a prerequisite for advancing biomedical research and facilitating clinical translational applications.

Transmission electron microscopy (TEM) is the most widely utilised technique for observing the morphology and structural characteristics of *F. nucleatum* OMVs. It provides high-resolution insights into the size, morphology, and architecture of OMVs. TEM images reveal that *F. nucleatum* OMVs are spherical vesicles and possess a double-layer membrane [17,34,45]. The underlying principle of TEM involves directing a focused electron beam through a thin specimen to generate an image of the sample [55]. This method, however, requires complex sample preparations, has limited detection throughput, and the staining during the sample preparation process may affect the structure of OMVs [56]. In addition, scanning electron microscopy (SEM) offers valuable insights into the three-dimensional surface structure of the OMVs [17,49,57].

Nanoparticle Tracking Analysis (NTA) employs laser scattering microscopic imaging technology to directly observe and track the Brownian motion of individual nanoparticles in suspension [58]. This technique simultaneously provides a detailed analysis of particle size distribution and concentration. In NTA, the sizes of *F.nucleatum* OMVs typically range from 40 nm to 200 nm. However, NTA imposes strict requirements on detection temperature, sample concentration, and operational procedures. Moreover, it is unable to distinguish between OMVs, bacterial fragments, or contaminants in the solution. In contrast, nano‑flowcytometry (FCM) differs from NTA in that it offers more rapid and higher-precision measurements of particle size and concentration for OMVs [17].

Various methods are available for analysing the composition of OMVs, including bicinchoninic acid (BCA) assay, SDS/PAGE, Western blotting, and mass spectrometry (MS). The BCA assay is a biochemical method used to determine the total protein concentration in a solution, indicated by a colour change of the sample from blue to purple, proportional to the protein concentration. It is the most commonly employed technique in the literature for quantifying protein concentrations in *F. nucleatum* OMVs [21,39]. SDS/PAGE can be used to identify the fractions containing OMVs during density gradient centrifugation [17]. Western blotting is utilised to confirm the enrichment of OMV-associated protein markers [45]. Furthermore, the purified OMVs are subjected to proteomic analysis using MS-based high-throughput techniques, which can identify thousands of proteins and provide evidence to reveal the biogenesis, pathogenicity, and antigenicity of OMVs [41,49].

### The constituents of *F. nucleatum* OMVs

OMVs are nano-sized particles produced by Gram-negative bacteria during the active growth stage. In recent years, an increasing number of studies have focused on the role of OMVs in bacterial pathogenicity, their interaction with host cells, and their effects on host organisms and the immune system [13]. Initially, OMVs were regarded merely as growth artifacts or by-products associated with cell lysis [59]. However, more and more researchers have realised that OMVs play a pivotal role in microbial virulence. Today, researchers utilise a range of analytical methods, such as mass spectrometry (MS) analysis, to investigate the composition, structure, and cargo-loading of OMVs.

The structural core of OMVs is composed of an asymmetric phospholipid bilayer. The outer leaflet is enriched with lipopolysaccharide (LPS), while the inner leaflet is predominantly composed of phospholipids, such as phosphatidylethanolamine [60]. This structure closely resembles the outer membrane of the parent bacterium. OMVs are primarily composed of proteins, lipids, and various pathogen-associated molecular patterns, including lipoproteins, peptidoglycan, and nucleic acids [61]. The luminal cargo of OMVs is predominantly composed of periplasmic proteins [62]. Proteins are among the most abundant components in OMVs and play diverse functional roles [63].

In one study, researchers conducted biophysical and proteomic analyses on purified OMVs from *F. nucleatum* subsp. animalis *7_1 (EAVG_002)*, which revealed the constituents and membrane fraction composition of OMVs [41]. Five inner membrane proteins were detected in the periplasmic and outer membrane fractions, suggesting these proteins may detach from the inner membrane, migrate to other fractions, and ultimately bind to OMVs. Three of these five proteins were classified as autotransporters. Outer membrane proteins were the most abundant component of OMVs, comprising 72% of the total protein, whereas 17% consisted of proteins with unknown subcellular localisation, 6% were identified as periplasmic proteins, and 5% were cytoplasmic proteins [41]. However, another study conducted proteomic analysis of OMVs derived from *F. nucleatum* ATCC 25586 and categorised the co-expressed proteins into six subtypes. The largest proportion of proteins belonged to the subtype cytoplasmic proteins, accounting for 62.8%, followed by uncharacterised proteins at 25.6%. Additionally, 3.5% of the proteins were cytoplasmic membrane proteins, 4.3% were outer membrane proteins, and 3.7% were periplasmic proteins [17]. These discrepancies may be attributed to variations in the *F. nucleatum* subspecies and the culture conditions employed. Moreover, FadA proteins, MORN2 domain-containing, and YadA-like proteins as putative virulence factors were also detected in OMVs [41], and these findings can be confirmed by a subsequent study [49].

Furthermore, pH influences the production levels and protein composition of OMVs from *F. nucleatum* ATCC 23726, although it has no significant influence on their morphology and size. Under acidic conditions, 70% of OMV proteins exhibited altered expression, with 306 proteins upregulated, including type 5a secreted autotransporter (T5aSSs) and 16 membrane occupation and recognition nexus (MORN) domain-containing proteins, and 360 downregulated. Additionally, MORN domain-containing proteins were found to be clustered near the genes of T5aSS autotransporter adhesin, suggesting a role in enhancing bacterial adhesion and invasion. In acidic OMVs (aOMVs) and neutral OMVs (nOMVs), a total of 991 proteins can be detected, including the known virulence proteins FadA, Fap2, FomA, and putative MORN2 domain-containing and Aim1 virulence proteins. These proteins were classified as outer membrane proteins, enzymes, and proteins involved in transport [49]. FadA is recognised as a virulence factor present in OMVs. However, a study has shown that a similar amount of purified recombinant FadA (rFadA) administered via intravenous injection in collagen-induced arthritis (CIA) mice does not exacerbate the severity of arthritis. This may suggest that the inherent surface of the lipid phase and physical structure of OMVs shield FadA from exposure to host clearance mechanisms, possibly by secreting antibodies, or that OMVs may contain certain active forms of FadA, which are not present in rFadA [21]. In addition, another study conducted the proteomic analysis of OMVs derived from *F. nucleatum*, revealing that FomA protein exhibited the highest relative abundance in OMVs, along with the strongest ability of pro-metastasis compared to other proteins [45].

OMVs, although lacking replicative or infectious capacity, possess pathogen-associated virulence factor antigenicity and have the ability to interact with host cells and immune cells. This interaction presents a potential avenue for the development of targeted vaccines [52]. In the future, more detailed proteomic and lipidomic studies could be conducted to elucidate the unique characteristics of OMVs derived from different *F. nucleatum* subspecies. Additionally, large-scale development of recombinant constructs may help reduce toxicity and optimise the efficacy of these vaccines.

### The role and mechanism of *F. nucleatum* OMVs in inflammatory disease

OMVs secreted by the parental bacteria exhibit pathogenicity comparable to that of the parental bacteria themselves. These vesicles act as vehicles for the long-distance transmission of virulence factors, which are involved in processes such as adherence, invasion, antimicrobial resistance, and modulation of host immunity. OMVs carry a variety of immunogenic molecules, such as lipopolysaccharide (LPS), FomA, and peptidoglycan, which activate the host innate immune system via Toll-like receptors (TLRs) [36,64,65].

Toll-like receptors (TLRs) are expressed on various innate immune cells, such as dendritic cells and macrophages, as well as non-immune cells, including fibroblasts and epithelial cells. These receptors are pivotal to the innate immune response, as they are germline-encoded pattern-recognition receptors (PRRs) responsible for the initial detection of microbes [64]. PRRs recognise microbe-specific molecular signatures, referred to as pathogen-associated molecular patterns (PAMPs). For example, TLR4 has been linked with the recognition of bacterial LPS [66], while TLR2 recognises peptidoglycan, lipopeptide/proteins, and outer membrane proteins FomA and OmpA [67].

This process is crucial for eliminating pathogens and virulence factors. However, an excessive immune response during inflammation can also result in tissue damage [63]. Recent studies have shown that PRRs on the membranes of both immune and non-immune cells recognise the PAMPs of *F. nucleatum* OMVs, triggering inflammatory responses. This interaction with host cells modulates the immune response, highlighting the role of OMVs in promoting inflammation and immune modulation (Figure 3).

**Figure 3.**
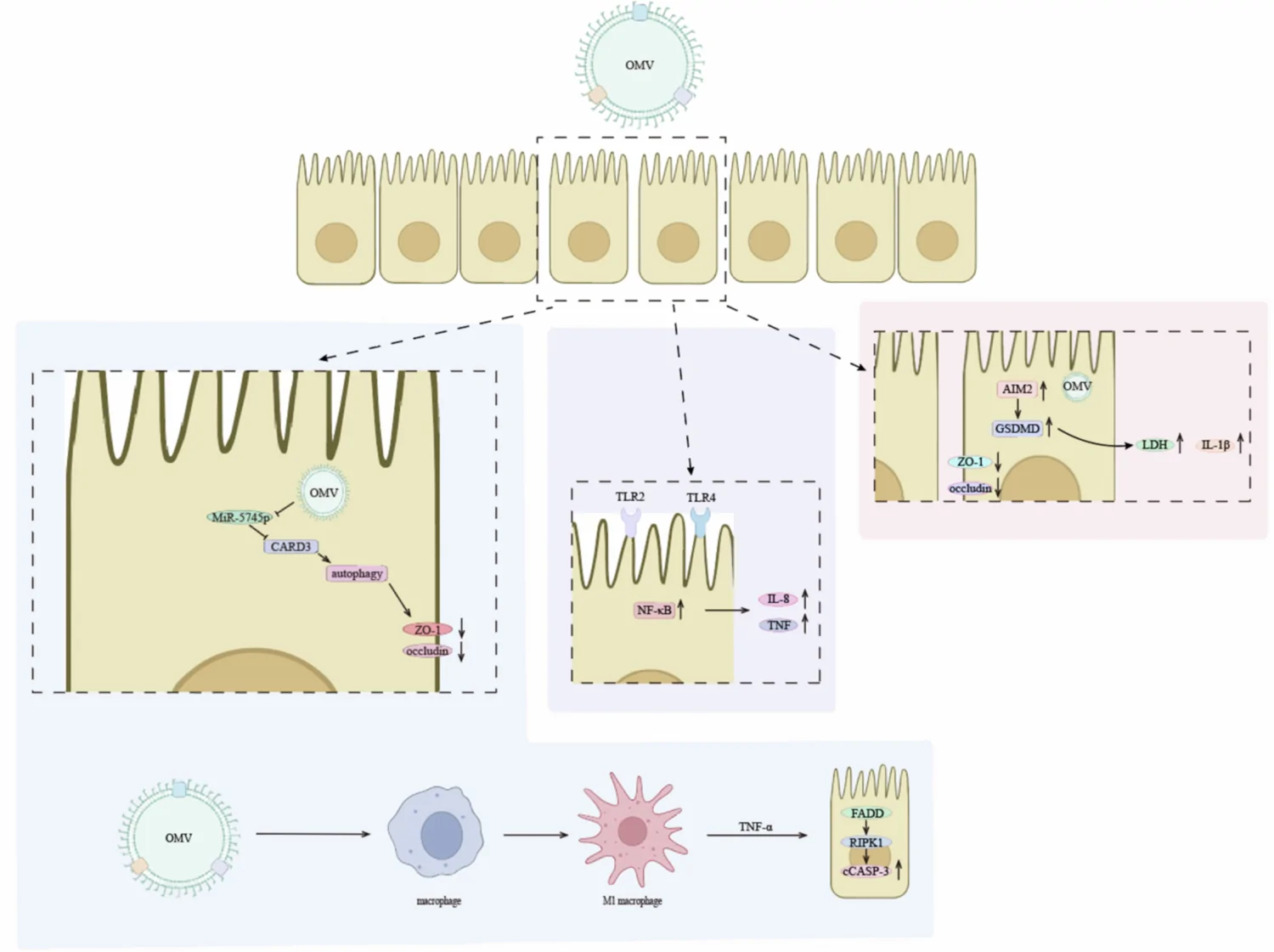
The possible mechanisms by which *F. nucleatum* OMVs contribute to inflammatory bowel disease. OMVs are internalised by intestinal epithelial cells and activate cell surface Toll-like receptors, leading to the downregulation of tight junction proteins. In addition, OMVs interact with immune cells, such as macrophages, thereby impairing intestinal barrier function.

#### The cascades of inflammatory pathway activation

Previous studies have shown that *F. nucleatum* accumulates in the faeces of patients with inflammatory bowel disease (IBD), and its abundance is related to the activity of the disease. In both cell lines and animal models, *F. nucleatum* downregulates the expression of tight junction proteins, such as zonula occludens-1 and occludin, thereby compromising epithelial integrity and increasing permeability. Moreover, *F. nucleatum* induces the secretion of various cytokines, including tumour necrosis factor-*α* (TNF-*α*), interferon-*γ* (IFN-*γ*), interleukin [IL]-1β, IL-6, and IL-17 [68]. Recent studies have demonstrated that *F. nucleatum* OMVs can independently function similarly with bacteria to mediate intestinal epithelial responses and inflammation, playing a crucial role in the pathogenesis of UC.

A study revealed that *F. nucleatum* subspecies *polymorphum* can adhere to intestinal mucus and secrete OMVs. And 25% *F. nucleatum* BHIS conditioned medium (>50-kDa fraction) and OMVs similarly increased the expression of IL-8 and TNF, as well as activated NF-κB signal transduction pathway in HT29 colonic cancer-derived cells. In contrast, TLR4 inhibitor CLI-095 significantly inhibited this effect, and conditioned medium (<50-kDa fraction) had no effect, similar to the negative control (BHIS). These results may suggest that the >50-kDa fraction contains OMVs, which induce epithelial inflammatory signals via TLR4 [35]. However, researchers also found that *F. nucleatum* did not adversely affect overall health parameters in a humanised mouse model with an intact human microbiota. This provides evidence that *F. nucleatum* promotes proinflammatory signalling cascades in vivo, but only in the presence of an antibiotic-disrupted humanised microbiota [35].

Moreover, several studies have proved that *F. nucleatum* OMVs can be internalised by intestinal epithelial cells (IECs) [48,50], triggering cellular inflammatory responses, including the upregulation of mRNA levels of IL-1β, IL-6, and TNF-*α* and the downregulation of mRNA levels of IL-10 and tight junction proteins ZO-1 and occludin. Similar experimental results have also been observed in an experimental colitis model [48]. Additionally, the expression of autophagy-related protein p62 was downregulated, while LC3II expression in the colon tissue of experimental colitis mice was upregulated, which can be reversed by using the autophagy lysosomal inhibitor, chloroquine (CQ). This demonstrates that OMVs may activate autophagy, leading to barrier dysfunction. Mechanistically, overexpression of miR-574-5p can rescue cellular inflammation, epithelial barrier damage, and autophagosome formation in vivo and in vitro models. However, this alleviating effect was dependent on the downstream target gene CARD3 of miR-574-5p. This may prove that OMVs exacerbate experimental colitis and activate autophagy depending on the miR574-5p/CARD3 axis [48]. In contrast, another study found that OMVs were not massively internalised into T84 intestinal epithelial cells after 4 h incubation [36]. They activated NF-κB signal pathway independent of TLR4 and LPS but relied on TLR2 and FomA, which have been proven to be present on OMVs. Further research revealed that OMV-mediated NF-κB signalling required dynamin and clathrin in T84 cells, rather than endocytosis [36].

Similar to previous studies [1,69,70], the abundance of *F. nucleatum* is associated with the severity of UC patients in colon biopsy tissues. Furthermore, higher expression of AIM2 was observed with moderate/severe UC compared to patients with mild UC. *F. nucleatum* activated AIM2 inflammasome in vivo and in vitro models, promoting UC progression by upregulating the expression of pyroptosis-related proteins GSDMD and IL-1β and the levels of serum lactate dehydrogenase (LDH), while downregulating tight junction proteins ZO-1 and occludin. Intriguingly, *F. nucleatum* OMVs were the primary inducers of AIM2 inflammasome activation in IECs to aggravate the progression of UC. However, this AIM2 inflammasome-dependent pyroptosis can be inhibited by *DNase I*, which degrades the DNA components in OMVs. This suggests that the DNA component in OMVs plays a pivotal role in compromising gut barrier function [50].

In addition, *F. nucleatum* OMVs entered into the human periodontal ligament stem cells (hPDLSCs) through endocytosis, primarily via caveolin-dependent endocytosis, which reduced the migration of hPDLSCs without affecting cell proliferation. Specifically, OMVs induce the activation of reactive oxygen species (ROS) and activate NLRP3 inflammasomes, leading to the upregulation of inflammatory factors, including IL-1β, IL-18, IL-6, and TNF-*α*, as well as extracellular matrix-degrading enzymes. Furthermore, OMVs impair the mineralisation capacity of hPDLSCs through NF-κB signalling activation, contributing to periodontium destruction and alveolar bone loss in periodontitis [17].

#### Modulating immune response

OMVs pass through the host’s epithelial cell barrier and enter the submucosa, where they can directly interact with various immune cells, including neutrophils, macrophages, and dendritic cells (DCs) [71]. And they can activate macrophage surface PRRs to modulate macrophage responses [72]. Specifically, *F. nucleatum* OMVs induce macrophage polarisation toward proinflammatory M1-like macrophages, inducing oxidative damage in cells, which results in the upregulation of ROS levels, production of proinflammatory cytokines such as TNF-*α* and inducible nitric oxide synthase (iNOS), and suppression of IL-10 expression [18,42].

Moreover, *F. nucleatum* OMVs promoted epithelial cell death in constructed macrophage/Caco-2 co-cultures in transwell plates, leading to the upregulation of the levels of LDH, FADD, RIPK1, and cCASP3, while inducing barrier damage by decreasing the ZO-1 protein levels. This effect was attenuated by the TNF-*α* neutralising antibody or Necrostatin-1 (Nec-1, a RIPK1 inhibitor) [42]. Furthermore, researchers found that immunoprecipitated RIPK1 was significantly enriched in RIPK3 in the *F. nucleatum* OMV-treated cells, and vice versa. This indicates that OMVs can promote RIPK1 and RIPK3 aggregation to form necrosomes in Caco-2 cells, eventually inducing necroptosis and damaging epithelial barrier function. Similarly, OMVs induced proinflammatory macrophages and activated FADD-RIPK1-cCASP-3 signalling pathway in experimental colitis mice, thereby weakening intestinal barrier function in UC [42].

*F. nucleatum* OMVs affected the apoptosis of mouse gingival fibroblasts (MGF) cells by stimulating the secretion of LDH, and they combined with M1 macrophages to exacerbate the release of LDH. Moreover, OMVs caused more extensive alveolar bone loss and density reduction in a mouse model of periodontitis, accompanied by an increase in the number of osteoclasts and elevated levels of IL-1b, IL6, and TNF-*α*. This indicates *F. nucleatum* OMVs and M1 macrophages exacerbate the occurrence and progression of periodontitis [18]. Intriguingly, further study indicated that OMVs derived from *F. nucleatum* could independently trigger the occurrence of periodontitis and increase the expression of CD11c (a marker of dendritic cells), CD3 (a marker of T cells), and F4/80 (a marker of macrophages) in the normal periodontal tissue of the rat model [17].

Rheumatoid arthritis (RA) is an autoimmune disorder characterised by elevated levels of serum inflammatory cytokines, including IL-6 and TNF-a, which trigger the inflammatory responses in the joints [73,74]. It was established that the abundance of *F. nucleatum* was positively correlated with RA disease activities, as well as serum inflammatory cytokines. In the collagen-induced arthritis (CIA) mouse model, *F. nucleatum* aggravated arthritis by secreting OMVs, which were internalised by macrophages to promote macrophage-associated inflammation. Furthermore, by constructing the ΔFadA-/- OMVs, researchers found that FadA in the OMVs mediated the pro-inflammation effects of *F. nucleatum* through Rab5a-YB-1 axis in bone marrow-derived macrophages (BMDM). And FadA-Rab5a-YB-1 axis was also abundant in synovium of RA patients. These results indicate that FadA-containing *F. nucleatum* OMVs may translocate from gut to joint, then target Rab5a-YB-1 axis to exacerbate RA by acting on synovial macrophages [21].

Additionally, the expression of ZBP1 was higher in the *F. nucleatum*-infected apical periodontitis (AP) tissues than in normal tissues, and this expression could be suppressed by siRNA, resulting in a significant decrease in the production of cytokines (IL1b, IL6, TNF, and Cxcl10). Specifically, *F. nucleatum* activated the expression of ZBP1 mRNA in the murine macrophage cell line RAW 264.7, leading to the increase of the protein expression of *N*-GSDME, cleaved-caspase 3, and pMLK. This suggests that *F. nucleatum* induces pyroptosis, apoptosis, and necroptosis in macrophages, which is consistent with the effect observed in macrophages treated with *F. nucleatum* OMVs. These findings indicate that *F. nucleatum* OMVs may induce a mixed mechanism of macrophage cell death in apical periodontitis, thereby contributing to the proinflammatory responses [75].

### The role and mechanism of *F. nucleatum* OMVs in carcinoma

*F. nucleatum* is a Gram-negative bacterium that has been identified as an important pathogenic agent associated with various types of cancer, such as oral squamous cell carcinomas (OSCC), colorectal cancer (CRC), and breast cancer (BC) [1,2,76]. A previous study using quantitative PCR analysis has confirmed that *F. nucleatum* is overabundant in human colorectal carcinoma tissues compared to normal control tissues and that this overabundance is positively associated with lymph node metastasis [77]. Moreover, OMVs secreted by Gram-negative bacteria are recognised as crucial mediators of communication between bacteria and host cells, and their roles in carcinogenesis and tumour development have gained increasing research attention in recent years. OMVs can move through the host circulatory system, involved in long-distance cargo transport to various organs [67]. Furthermore, compared to the normal intestine, the pH value of the tumour microenvironment is weakly acidic. Therefore, a study investigated the changes in OMVs derived from *F. nucleatum* ATCC 23726 under acidic conditions similar to the tumour microenvironment, revealing the increase in OMV production levels and alterations in the protein composition [49]. Thus, it is crucial to elucidate the role of OMVs in the host-bacteria interaction mechanism involved in cancer (Figure 4).

**Figure 4.**
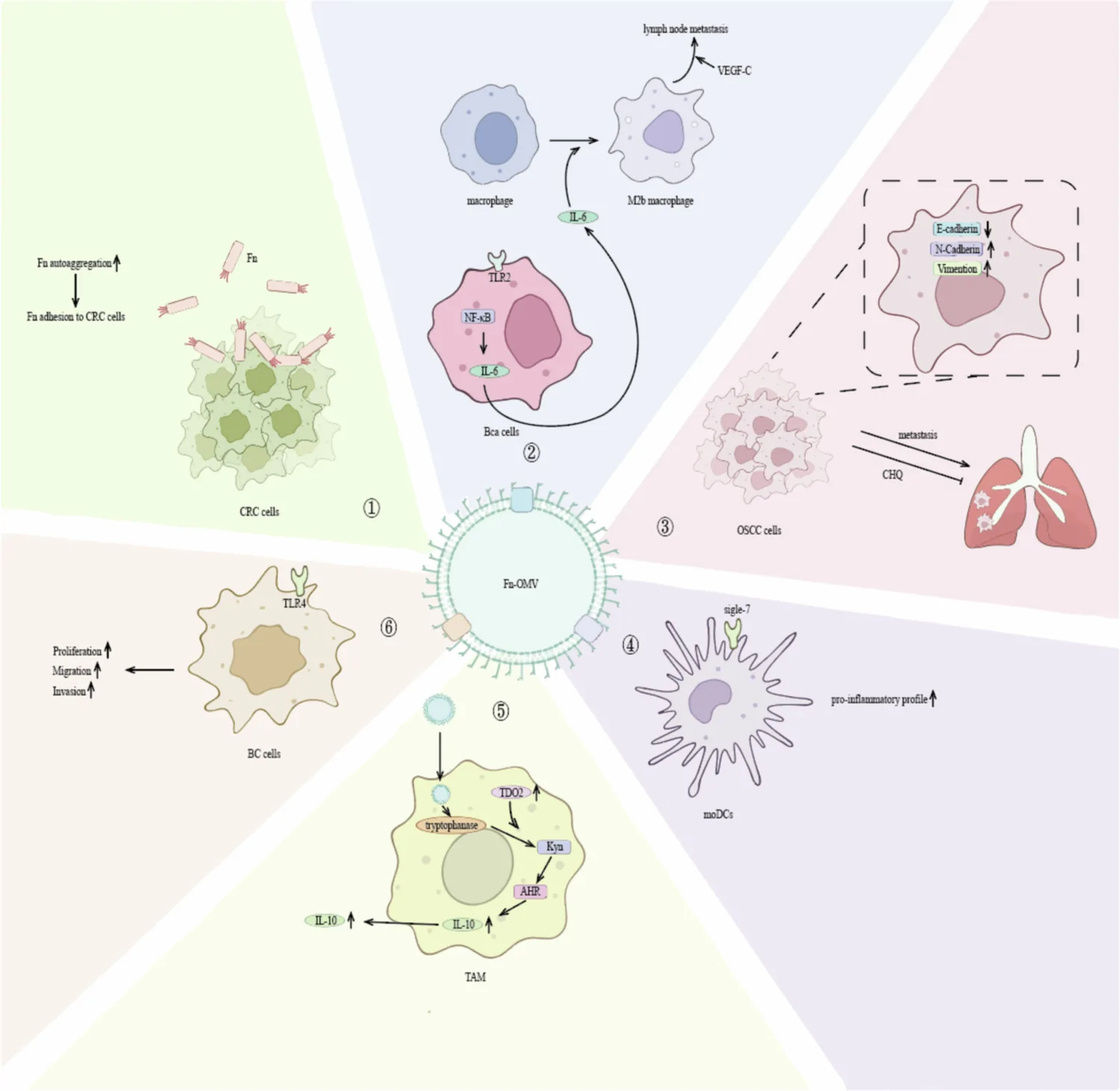
The role of *F. nucleatum* OMVs in cancer development and metastasis. (1) OMVs promote the *F. nucleatum* autoaggregation and *F. nucleatum* adherence to CRC cells. (2) OMVs activate the TLR2/NF-кB/IL-6 axis in BCa cells and induce IL-6–dependent macrophage polarisation toward an M2b phenotype, facilitating lymph node metastasis. (3) OMVs induce epithelial–mesenchymal transition in OSCC cells to promote lung metastasis. (4) OMVs interact with Siglec-7 on moDCs surface triggering a proinflammatory response. (5) OMVs containing tryptophanase induce the TDO2/AHR axis in TAMs, contributing to an immunosuppressive environment in HNSCC. (6) OMVs activate TLR4 pathway to enhance the proliferation, migration, and invasion of BC cells. Abbreviations: CRC, colorectal cancer; BCa, bladder cancer; BC, breast cancer; OSCC, oral squamous cell carcinomas; moDCs, monocyte-derived dendritic cells; TAMs, tumour-associated macrophages; CHQ, chloroquine.

#### Adhesion, progression, and metastasis

A study revealed that *F. nucleatum* OMVs were enriched in clinical CRC tissue. These OMVs promoted *F. nucleatum* adherence to CRC cells, leading to bacterial colonisation and exacerbating CRC progression. This adhesion was dependent on the membrane fusion between OMVs and recipient CRC cells, as well as on the OMV surface protein FomA through its interaction with another surface protein FN1441, which also promoted *F. nucleatum* autoaggregation [51].

Furthermore, *F. nucleatum* OMVs enhanced Caco-2 cell migration, but active compounds from the fruits of *P. polyphylla* (pennogenin 3-O-beta-chacotrioside, polyphyllin VI) and saponin I inhibited migration. However, these compounds also suppressed the growth of *Bifidobacterium bifidum*, a common probiotic in human gut microbiota. This indicates supplementation with *Bifidobacterium bifidum* is necessary when these compounds are used as a complement to CRC treatment [40]. Moreover, *F. nucleatum* was overabundant in BC tissues compared with normal breast tissues, and the expression levels of *F. nucleatum* gDNA were significantly associated with tumour size and metastasis. *F. nucleatum* OMVs enhanced the malignant behaviours of BC cells, including proliferation, migration, and invasion, which may be attributed to the activation of TLR4 pathway [38].

Furthermore, another study revealed that *F. nucleatum* OMVs promoted lung metastasis of OSCC xenografts, probably because they induced the EMT phenotype, which plays a crucial role in cancer metastasis in cancer cells, through the expression of P62 and E-cadherin downregulation and LC3-II, *N*-Cadherin, and Vimentin upregulation. CHQ (chloroquine, an autophagic lysosomal inhibitor) attenuated EMT in cancer cells. This suggests that OMVs promote tumour metastasis through autophagy [34].

#### Modulating immune response

A study revealed that there may be an interaction between *F. nucleatum* ATCC 51191 and Siglec-7 on U-937 cells’ surface. *F. nucleatum* OMVs in which LPS has been identified as a main constituent by GC-MS from ATCC 51191 bind to Siglec-7 at levels comparable to LPS extracted from *F. nucleatum*. Moreover, *F. nucleatum* and derived components (OMVs and LPS) promoted a proinflammatory profile in human monocyte-derived dendritic cells (moDCs), while *F. nucleatum* induced a tumour-associated profile in macrophages (moMфs and U-937 cells). And Siglec-7 was involved in the induction of proinflammatory response in moDCs by *F. nucleatum* ATCC 51191. This demonstrates that Siglec-7 plays a key role in mediating immune modulation by *F. nucleatum* strains and their OMVs through recognition of LPS on the bacterial cell surface [37].

Further studies have shown that the abundance of *F. nucleatum is* significantly higher in HNSCC tissues than in normal tissues, and the elevated levels of *F. nucleatum* increase the risk of larger tumour sizes, advanced TNM stage, and metastasis. Nevertheless, *F. nucleatum* OMVs did not promote the growth of HNSCC cell lines in vitro but instead exacerbated HNSCC by creating an immunosuppressive environment through manipulation of tumour-associated macrophages (TAMs). Specifically, *F. nucleatum* OMVs contained tryptophanase, which induced indole production in macrophages, which subsequently upregulated the expression of TDO2, leading to the activation of AHR expression through kynurenine products. This created an immune suppression niche, followed by inhibiting the infiltration of CD8^+^ T cells and CTLs while promoting the infiltration of Tregs. This study reveals that the TDO2/AHR axis plays an important role in *F. nucleatum* OMVs mediating pro-tumorigenesis and their inhibitory impact on immunotherapy [39].

Similarly, the abundance of *F. nucleatum* in bladder tumour tissues is higher compared with adjacent normal tissues, and it is elevated in bladder cancer (BCa) patients with lymph node involvement LN (+) groups relative to those in LN (–) groups. Moreover, *F. nucleatum* promoted lymph node metastasis of BCa mainly through the secretion of OMVs, which depended on FomA, a major protein component of OMVs, activating the TLR2/NF-κB/IL-6 signalling axis in BCa cells. This subsequently induced IL-6–dependent polarisation of macrophages toward an M2b phenotype, thereby enhancing the expression of VEGF-C (a key regulator of lymphatic vessel formation and lymph node metastasis). However, FomA-TLR2 interaction can be suppressed by pinocembrin to inhibit BCa cell migration and metastatic progression, indicating the potential of pinocembrin as a therapeutic agent for BCa [45].

### The therapeutic potential of *F. nucleatum* OMVs

The surface of OMVs possesses abundant PAMPs, which are beneficial for the recognition and connection with antigen-presenting cells. OMVs are recognised as nano-sized biological vesicles to enhance the efficacy of cancer treatment and combat cancer drug resistance due to their superior biocompatibility, natural drug-loaded capacity, and relatively easy to engineer [30]. Because of these properties, OMVs have enormous potential and advantages for being designed as drug delivery vehicles, targeting specific cells or tissues while maintaining drug activity. Moreover, in recent years, some studies using biomimetic nanoparticles based on OMVs, which mimic bacterium interactions, have been involved in various therapies, including immunotherapy, anticancer treatments, and antibacterial applications [78,79].

In a study, researchers constructed an OMV-coated nanoplatform (OMV@Fe-ZIF-90@M nanoparticles) by embedding metronidazole (MTD) into bimetallic metal organic frameworks and coating them with *F. nucleatum*-derived OMVs. OMV@Fe-ZIF-90@M was easily internalised by *F. nucleatum* and effectively suppressed it. Moreover, this nanoplatform efficiently targeted triple-negative breast cancer (TNBC) with the help of OMVs, leading to significant suppression of *F. nucleatum*-colonised tumours, and demonstrated sufficient safety for bioapplication in healthy Balb/c mice. OMV@Fe-ZIF-90@M treatment induced immunogenic cell death (ICD) with antibacterial ability, stimulating dendritic cell (DC) maturation, secretion of proinflammatory cytokines, and T-cell infiltration, thereby converting a ‘cold’ tumour immune microenvironment into a ‘hot’ one to enhance anti-TNBC therapy [43].

A recent study constructed a novel biomimetic nanoparticle (FMV@PCFPC) by loading *F. nucleatum* OMVs and antibacterial drug cinnamaldehyde (CA) into PCFP, which was designed using a host-guest complex of hemirotaxane (mPEG-*β*-CD) and polyethyleneimine-ferrocene (Fc-PEI). These components were coated to form the drug delivery system. This system exhibited high stability, biosafety, targeting capability, and H_2_O_2_-responsive release, mainly relying on cinnamaldehyde and Fe^2+^ in PCFPC, which also inhibited biofilm. Furthermore, FMV@PCFPC entered tumour cells to clear intratumoral bacteria and exerted antibacterial and antitumor effects via the ferroptosis pathway. It also activated ICD, resulting in DC maturation and promoting cytotoxic T cell infiltration and cytokine secretion, therefore paving the way for effective cancer immunotherapy [46].

In addition, PANoptosis is an inflammatory cell death mediated by cytokines and plays a significant role in tumour treatment by triggering a robust immune response [80,81]. Herpes simplex virus-1-based oncolytic viruses (oHSV) induced the accumulation of Z-RNA, thereby increasing ZBP1-mediated PANoptosis in tumour cells, exhibiting anti-tumour effects. *F. nucleatum* OMVs enhanced the expression of the PANoptosis executor proteins gasdermin D/E (GSDMD/E) and mixed lineage kinase domain-like protein (MLKL) by affecting protein ubiquitination. Further research revealed that *F. nucleatum* OMVs increased the expression of PANoptosis executor proteins, thereby enhancing the ability of oHSV to induce PANoptosis in tumour cells. Moreover, the combination of OMVs and oHSV increased CD8^+^ T cell infiltration, inflammatory markers in the tumour microenvironment, and PD-L1 upregulation while decreasing regulatory T (Treg) cells and enhancing the response to immune checkpoint blockade (ICB), thereby converting ‘cold’ tumours into ‘hot’ tumours and improving anti-PD-L1 immunotherapy [47].

In another study, researchers developed OMV-ABSI nanospheres, which were NIR-triggered dual PDT/PTT nanospheres, AgBiS_2_@ICG (ABSI), coated with *F. nucleatum* OMVs. These nanospheres attached to the bacterium surface through the adhesin interactions, thereby maximising the antimicrobial effect of PDT/PT. Meanwhile, exogenous ROS and heat caused damage to the outer membrane, protein, and lipid, resulting in a portion of bacterial death. When this primary damage was insufficient to directly cause bacterial death, it induced oxidative stress, accelerating ETA, and promoting the accumulation of endogenous ROS. This, in turn, inhibited DNA transfer and repair, resulting in secondary damage and ultimately leading to bacterial death. In the in vivo periodontitis treatment model, the nanospheres reduced multispecies bacterial colonisation by nearly 3 logs, inhibited osteoclast formation, and transitioned active periodontitis into a more quiescent state [44].

However, engineered OMV-based nanoplatforms have emerged as promising next-generation drug delivery systems because of their stable payload capacity, unique nanoscale structure, and excellent biocompatibility [82]. Nevertheless, safety concerns remain before their clinical translation [83]. In particular, *F. nucleatum* OMVs naturally contain virulence-associated components, including proinflammatory and potentially oncogenic molecules, which may induce excessive immune activation and unintended pathological effects. Therefore, future engineering strategies should not only enhance antigen presentation and targeting efficiency but also minimise the toxicity associated with endogenous OMV components [84]. Rational engineering approaches, such as proteomic remodelling to remove or attenuate virulence factors, endotoxin detoxification, and the use of non-pathogenic bacterial strains or genetically engineered endotoxin-free chassis, may substantially improve the biosafety profile of OMV-based therapeutics [85]. These strategies are expected to preserve their therapeutic efficacy while reducing off-target inflammatory responses, thereby facilitating their safe clinical translation.

## Conclusion

*F. nucleatum* OMVs are critical mediators of bacterial pathogenesis, possessing the ability to transport virulence factors, including FadA, Fap2, FomA, and MORN2 domain-containing proteins, highlighting their key role in interactions between bacteria and host cells. FomA can mediate the interspecies adhesion of *F. nucleatum* and remains on the CRC cell surface, facilitating bacterial adhesion. This may serve as a potential vaccine target to inhibit bacteria autoaggregation and aid cancer therapy or as a biomarker for diagnosis. In conclusion, *F. nucleatum* OMVs can be recognised by the TLR receptors to induce the production of proinflammatory cytokines and activate inflammatory responses. Moreover, *F. nucleatum* OMVs, as nanoscale particles, can induce a tumour-immunosuppressive microenvironment, promoting the progression and metastasis of cancer. In addition to their pathogenic effects, the potential of engineered OMVs provides new insight into biomedical applications in antibacterial therapy, as well as cancer immunotherapy by modifying the immunosuppressive tumour microenvironment.

## Challenges and future research directions

In this review, we have found that the isolation and purification process of *F. nucleatum* OMVs involves multiple steps and is time-consuming. The purified OMVs exhibit heterogeneity in protein composition and particle numbers due to the variations in the parent bacterium, cultivation conditions (growth phase, pH, temperature, and medium composition), and isolation techniques. As a result, it is difficult to conduct standardised assessments of the pathogenic response of OMVs due to the use of different concentrations and methodological variations across studies. Consequently, it is essential to develop standardised protocols for OMVs production, isolation, purification, and quantification to enhance the comparability among studies and improve the reproducibility of the research. Only in this way can the studies on OMVs be comparable and lead to precise conclusions regarding OMV-host cell interactions.

Additionally, numerous studies have explored the pathogenicity of *F. nucleatum* OMVs in various diseases and therapeutic potential. However, most of these studies are in the initial stages of the research, using cell lines and animal models, which results in less convincing evidence. Notably, no eligible clinical studies investigating *F. nucleatum* OMVs were identified in this review. Several methodological barriers may explain this gap. As discussed above, OMVs exhibit considerable heterogeneity in size and composition, even when isolated from pure bacterial cultures. Moreover, the physical separation of different OMV subtypes from biological fluids remains technically challenging [28]. Accurate detection and quantification of *F. nucleatum* OMVs in clinical specimens (e.g. gingival crevicular fluid, saliva, and serum) are further hindered by their nanoscale size, biological heterogeneity, and the overwhelming abundance of host-derived extracellular vesicles (EVs), including exosomes and microvesicles, which share similar physicochemical characteristics [86]. Consequently, clinical studies investigating the pathogenic roles and underlying mechanisms of *F. nucleatum* OMVs remain scarce, whereas existing clinical research has primarily focused on bacterial EVs as diagnostic biomarkers [87].

Furthermore, because OMVs contain components from the *F. nucleatum*, it is difficult to identify specific markers that distinguish between the bacterium and its OMVs in clinical samples. Therefore, it is essential to develop effective tools to elucidate the interaction between *F. nucleatum* OMVs and related diseases in clinical trials. Many challenges in OMV research remain unresolved, and many basic and clinical studies are still needed before OMVs can be used for disease diagnosis, treatment, and prevention. Future translational studies employing standardised methodologies and clinically representative cohorts are needed to bridge the gap between preclinical studies and clinical applications.

Moreover, unlike the well-established role of *Porphyromonas gingivalis*-derived OMVs in both oral and systemic pathologies, current investigations into *F. nucleatum* OMVs are predominantly concentrated on intestinal inflammation diseases and colorectal tumours. The interactions between *F. nucleatum* OMVs and various types of host cells remain underexplored and warrant further investigation in the future. This distribution likely reflects the well-established association between *F. nucleatum* colonisation and colorectal carcinogenesis, as well as the growing interest in the gut microbiome. However, such a narrow research focus may limit our understanding of the broader biological functions of OMVs in other disease settings. Increasing evidence suggests that *F. nucleatum* also contributes to the pathogenesis of atherosclerotic cardiovascular diseases, Alzheimer’s disease, oral squamous cell carcinoma, adverse pregnancy outcomes, and other systemic diseases [1,88], yet the roles of OMVs in these conditions remain insufficiently investigated. Expanding future research beyond CRC and intestinal inflammation would provide a more comprehensive understanding of OMV-mediated host–microbe interactions and facilitate the development of broader diagnostic and therapeutic applications.

## Data Availability

Not applicable.
